# 301 Youtube as a Source of Information for Skin Graft Procedures

**DOI:** 10.1093/jbcr/irad045.276

**Published:** 2023-08-29

**Authors:** Grant B Torres, Ludwik K Branski, Kimberley C Brondeel, Sai Kamma, Bradley Nus, Trey Sledge, Kylie Wu

**Affiliations:** University of Texas Medical Branch, Galveston, Texas; Shriners Children’s- Texas, University of Texas Medical Branch, Galveston, Texas; University of Texas Medical Branch, Galveston, Texas; University of Texas Medical Branch, Galveston, Texas; University of Texas Medical Branch, Galveston, Texas; University of Texas Medical Branch, Galveston, Texas; UNT Health Science Center, Fort Worth, Texas

## Abstract

**Introduction:**

YouTube, one of the most popular sites on the internet, has become a primary source of healthcare information for patients. Although YouTube videos about skin graft procedures have accrued millions of views, there lacks a publication defining their educational quality. With recent articles revealing the misleading nature of healthcare information on YouTube, this study aims to evaluate the quality of videos pertaining to skin graft procedures.

**Methods:**

YouTube was searched for various terms such as “Skin Graft Procedures” and “Skin Graft Surgery” with content stratified by total view count. After applying exclusion criteria, the top 84 most viewed videos were assessed, 24 of which implicating burn injuries. Four independent reviewers rated the material with the Global Quality Scale (GQS; 5 = highest quality, 1 = lowest quality) to judge educational value based upon factors such as publisher credibility, delivery of accurate information, and presence of biases. Viewership, source, educational modality, and date of upload were also collected from each video and compiled for further analysis.

**Results:**

The mean GQS score of all videos was 2.60. Videos uploaded by physicians (80.1%) had a significantly higher average GQS score than those not uploaded by physicians (p < 0.01). When grouped by modality, patient-friendly delivery methods (animations, news reports, etc.) contained the lowest quality information, while physician-led presentations provided the highest educational value (p < 0.01). Assessment of videos split into cohorts based on viewership noted a significantly higher GQS in videos with lower view counts (p < 0.05). There was no correlation between upload date and quality.

**Conclusions:**

Overall, skin graft videos found on YouTube provide low quality information. This is especially true for the “popular” videos, where an inverse correlation was found between view count and GQS score. Likewise, patient-friendly videos received the lowest GQS scores amongst the cohorts. Alternatively, videos performed by physicians, most notably physician-led presentations, significantly improved the educational quality of videos pertaining to skin graft procedures. As such, further analysis of skin graft videos found on YouTube could poise physician involvement in producing videos of high educational quality while potentially bridging the gap spanning quality content and video popularity.

**Applicability of Research to Practice:**

With online platforms such as YouTube evolving into conventional sources of healthcare information, physicians must act to improve the quality of online content to better guide patients in navigating treatment options and making healthcare decisions.

